# Pharmaceutical Advances and Proteomics Researches

**DOI:** 10.22037/ijpr.2020.112440.13758

**Published:** 2019

**Authors:** Ensieh KhalKhal, Mostafa Rezaei-Tavirani, Mohammad Rostamii-Nejad

**Affiliations:** a *Proteomics Research Center, Faculty of Paramedical Sciences, Shahid Beheshti University of Medical Sciences, Tehran, Iran. *; b *Gastroenterology and Liver Diseases Research Center, Research Institute for Gastroenterology and Liver Diseases, Shahid Beheshti University of Medical Sciences, Tehran, Iran.*

**Keywords:** Proteomics, Pharmacology, Medicine, Biomarker

## Abstract

Proteomics enables understanding the composition, structure, function and interactions of the entire protein complement of a cell, a tissue, or an organism under exactly defined conditions. Some factors such as stress or drug effects will change the protein pattern and cause the present or absence of a protein or gradual variation in abundances. The aim of this study is to explore relationship between proteomics application and drug discovery. "proteomics", "Application", and "pharmacology were the main keywords that were searched in PubMed (PubMed Central), Web of Science, and Google Scholar. The titles that were stablished by 2019, were studied and after study of the appreciated abstracts, the full texts of the 118 favor documents were extracted. Changes in the proteome provide a snapshot of the cell activities and physiological processes. Proteomics shows the observed protein changes to the causal effects and generate a complete three-dimensional map of the cell indicating their exact location. Proteomics is used in different biological fields and is applied in medicine, agriculture, food microbiology, industry, and pharmacy and drug discovery. Biomarker discovery, follow up of drug effect on the patients, and in vitro and in vivo proteomic investigation about the drug treated subjects implies close relationship between proteomics advances and application and drug discovery and development.

This review overviews and summarizes the applications of proteomics especially in pharmacology and drug discovery.

## Introduction and history

Proteins, the polymers of amino acids, play important roles in cellular metabolic activities. Cells respond to internal and external changes by regulating the activity and level of their proteins. Changes in the proteome (a collection of all the proteins in the body) provide a snapshot of the cell activities and physiological processes and they would describe the living cell dynamics. Proteomics, the large-scale study of proteins, enables understanding the composition, structure, function and interactions of the entire protein complement of a cell, a tissue, or an organism under exactly defined conditions (1). 

Emil Fischer and Franz Hofmeister, reported about proteins in 1902 and The term “protein” was initially introduced in 1938 by the Swedish chemist Jöns Jakob Berzelius. In 1975 the first protein studies that can be named proteomics was begun with the introduction of the two-dimensional gel and mapping of the proteins from the bacterium *Escherichia coli*, guinea pig and mouse. Many proteins could be separated and visualized but they could not be identified. In the early 1990s the terms “proteome” and “proteomics” were coined by Marc Wilkins (2, 3).

Proteome analysis gives a much better understanding of an organism than genomics because genomics can give a rough estimation of expression of a protein. Most of the proteins function are in collaboration with other proteins, and proteomics identifies which proteins interact so it is often considered as the advanced step in the study of biological systems because an organism’s genome is more or less constant and the total protein expression profile always changes with time, micro and macro environmental condition (4).

Based on the protein response, proteomics is classified into different groups: 

1-Structural proteomics that can help to understand three dimensional shape and structural complexities of functional proteins, can give detailed information of the structure and function of protein complexes present in a specific cell or organism, and can predict the structure of a protein when its amino acid sequence is determined directly by sequencing or from the gene with a method called homology modeling. X-ray crystallography and NMR spectroscopy were mainly used for structure determination (5).

2-Expression proteomics that is used to study the qualitative and quantitative expression of total proteins under two different conditions (the normal cell and treated or diseased cell) and investigates the expression protein patterns in abnormal cells. 2-D gel electrophoresis and mass spectrometry technique (MS) were used to determine the protein expressional changes. Identification of these proteins expressional changes will give valuable information about molecular biology of disease-specific manner for use as diagnostic markers or therapeutic targets (6, 7).

3-Functional proteomics that determines the protein functions, demonstrates how proteins assemble in larger complexes, and identifies the interacting protein partners. Using functional proteomics, an unknown protein with partners belonging to a specific protein complex involved in a particular mechanism can be identified its biological function (8, 9).

The modifications in the protein expression such as stress or drug effects will change the protein pattern and cause the present or absence of a protein or gradual variation in abundances. The main goal of proteomics is not only to pinpoint whole of the proteins in a cell, but also to generate a complete three-dimensional map of the cell indicating their exact location. Furthermore, proteomics analyzes and compares protein expression profiles, and determines where and in which ratio and under what condition proteins are expressed. It shows the observed protein changes to the causal effects (10).Understanding the structure, function and modification of each protein and the complexities of protein-protein interactions will be critical for developing the most effective diagnostic techniques and disease treatments in the future. An important use of proteomics is using specific protein biomarkers to diagnose disease in medicine (11). Proteomics is the method that has high impact in the economy of pharmaceutical industry via biomarker discovery, follow up of patients, evaluation of drug efficacy in the treated samples, and also capacity for joint to the other large scale methods as like bioinformatics. There for in the present study application of proteomics in the biomedical fields especially drug discovery and development is reviewed.

## Methods

PubMed (PubMed Central), Web of Science, and Google Scholar were searched for full text articles on the associated keywords “proteomics”, “Application”, and “pharmacology” published until August 2019. After title screening and study of the selected abstracts, 118 documents counting the selected ones and the related individuals were included. 

*Application of proteomics*

Today protein-protein interaction, protein function, modifications, and localization studies are explored by proteomics for advancement in science, technology and industry. Proteomics is used in different biological fields and is applied in medicine especially in oncology, bio-medicine, agriculture, food microbiology and industry, and pharmacy and drug discovery. In the following sections application of proteomics in clinic and medicine, Agriculture, nutrition and food industry, and pharmaceutical industry is discussed. 

*Clinic and Medicine*

Proteomics is used in clinic and medicine for translation new discoveries and technologies into improving patient outcomes, development of understanding of biochemical processes underlying diseases etiology. Proteomics tools have a key role in clinical chemistry, particularly for quantifying metabolites and hormones. The development of targeted proteomics methods support clinical studies specially handling complexities of tissues and bodily fluids, and generate new clinical tests from biomarker discovery (12, 13). In this regard; proteomics studies in the fields of oncology, cardiovascular diseases, neurodegenerative diseases, kidney disease and endocrine disease and disorders are summarized as follow:

*Oncology*

 Characterizing the protein expressions and functions of tumor cells causes to discover biomarker for prediction outcomes during cancer treatment and management. Description of the molecular and cellular mechanisms underlying tumor metastasis is the major challenge in medicine therefore the protein expressions analysis that is correlated to the metastatic process helps to understand the mechanism of metastasis and thus facilitate the development of strategies for the therapeutic interventions and clinical management of cancer (14). In the other hand early detection is an important trend in the cancer management. There are several evidences that proteomics was applied to introduce early diagnosis biomarker related to the cancers such as breast, colon, stomach, lung, and brains cancers (15-17).

*Cardiovascular diseases*

The main advantage of targeted proteomics methods is biomarker development in disease applications such as cardiovascular disease. By using targeted proteomics, putative markers for treatment of hypertrophic cardiomyopathy and myocardial infarction are determined (18).

 Several quantitate cardiovascular disease biomarkers such as LDH-B, CKMB, myoglobin, and troponin I, in serum samples from myocardial infarction patients can be measured accurately by proteomics tools (19). Identification of disease-specific proteins for dilated cardiomyopathy and anti-endothelial cell antibodies as a potential predictive test for chronic heart transplant rejection is investigated by using proteomics studies and databases (20-22).

*Neurodegenerative diseases and disorders*

A number of major proteins have been linked to neurodegenerative disorders. Many their molecular function are unclear. To understand their functions, the identification binding partners of these proteins by using quantitative interaction proteomics is necessary. Some studies show that interaction partners are linked to phenotypes of disease in vivo. By proteomic tools, wild-type proteins and disease-associated variants involved in pathogenesis of neurodegenerative diseases, are identified e.g for Alzheimer’s disease Amyloid beta precursor protein (APP) and Presenilin-1 (PSEN1); for Huntington’s disease, Huntingtin (HTT), for Parkinson’s disease, Parkin (PARK2) for spinocerebellar ataxia type 1, and Ataxin-1 (ATXN1) are known. Based on these principles, detection of amyloid beta in CSF was proposed for clearing delineation of Alzheimer’s disease from other disorders. Also the presence / absence of tau fibrillation and measured by PET or phosphorylated-tau in CSF can classify Alzheimer’s disease (23).

*Kidney*

The kidney is built from proteins and its damage is preceded and determined by protein changes. Proteins are key in maintaining kidney function. A significant change of the protein level causes chronic kidney disease. To improve knowledge on the molecular pathophysiology, early detection and prog-nosis, and management of chronic kidney disease, proteome analysis has been applied in multiple studies. By proteomic studies, a number of biomarkers for patient management are identified. In addition, proteome analysis identifies the best suited therapeutic target (24-26).

*Endocrine diseases and disorders*

Endocrine diseases such as hypothyroidism, Adrenal disease and diabetes, show abnormalities in many different organs and tissues. To detect of proteins related to the diseases is necessary for understanding of the pathophysiologic mechanisms that underlie disease and identifying accurate predictive, diagnostic and therapeutic biomarkers. through quantitative and qualitative proteome analyses, biomarkers of diabetic retinopathy in the plasma (27) and serum (28), Niemann-Pick (29), Fabry disease (30) (31), Gaucher disease (32) inborn errors of metabolism (33), Graves’ disease (hyperthyroidism) (34, 35), Cushing syndrome (Adrenal disease ) (36), and other endocrine diseases have been sought..

*Bio-medical Sciences*

 The study of interactions between microbial pathogens and their hosts, the infections origin and their effect on organs is an important area in proteomics. Prevention and curetting of disease at starting level is the main aim in the proteomics research. Proteomics can help to discover diagnostic markers and vaccine candidates, understand pathogenic mechanisms and expression patterns in response to different stimuli. Depending on the functional protein networks, mechanism of action of antibiotics in treating bacterial diseases can be understood and development of new antibiotic can be done (37, 38). 

*Agriculture*

The study of plant protein networks and plant traits mechanisms such as resistance to stresses help to sustain of agricultural crop production through protecting crops against biotic and abiotic stresses underlying the development and vitality of seeds. Some factors including high temperature, salt and heavy metal stress, and pathogens cause serious problems for agricultural management and reduce grain yield and quality. The use of proteomics in agricultural is presented especially for analyzing the interaction between crops and bacteria, and the regulation of male sterility represents a major target in crop, also it identifies proteins that are accumulated in organs under of biotic and abiotic stresses and identification of these proteins play crucial roles in plant growth and seed regulation (39-44).

*Food Microbiology*

Proteomics is used in food technology and food biotechnology for characterization and standardization of raw materials, process development and validation of downstream processing, determination of batch-to- batch variations and quality control of the final product. It is also used as a tool for the detection of possible bacterial contamination and microbial safety (45, 46).

Also it has been successfully applied to the study of food protein groups such as milk, meat, and Cereal proteins by many proteomic researches, and significant effort has gone into the characterization of their proteomes. Proteomics approaches have been used to compare milk proteins from different species and other dairy products, deplete, fractionate ,or remove some proteins of milk, and discover and characterize of bioactive milk components (47-51) .

Using proteomic approach, the insoluble protein fraction of meat and changes during meat aging in tough and tender beef were examined, the myofibrillar muscle fraction is analyzed, markers for muscle hypertrophy were identified, and the degree of protein degradation and meat quality were determined (52-56). Characterization of post-mortem changes in the various species of fish, the effect of additives during the processing of fish muscle, understanding the effects of environmental, nutritional, biological and industrial factors on fish meat quality were determined by using proteomic approaches (57-59) .

The construction of a rice proteome database including major proteins involved in growth and stress response was described. Also the proteomic profile of rice bran with a view to understanding its functional properties was studied, and how rice leaves respond to various environmental factors was determined. Other cereals include barley, maize, wheat have been examined using proteomics (60-66).

*Pharmaceutical industry*

In the past it was assumed that the sequencing of the human genome would result in detection of new drug targets and subsequently lead to more efficient and safer drugs. By using genetic methods, potential novel drug targets were identified; it soon became apparent that for assessing the suitability of a target for pharmacological intervention, information provided by the genomic is not sufficient. 

In human, a proteome consists out of about 100 000 proteins while there is a set of 10 000 genes for a cell type-specific expression. There are the enormous variability in the composition of a proteome and the ability to form an infinite number of phenotypes (67). Genetics has been determined gene function and has been used to extrapolate protein function and to understand the molecular basis of disease (68). Because of they do not provide relevant pharmacological information on the target, genetic may be limited. The modifications at the gene level do not reflect how a drug will work in comparison to direct modulation of the protein product. 

Proteins are the main executors of biological function and are greater in number, dynamics and complexity than genes so they generate the basis for the functional diversity in living systems and provide the classical molecular target for drug intervention at the same time.

Some environmental factors such as stress or drug affect the modifications of the expression parameters to cause the presence or absence of a protein or gradual variations in abundances and change the protein pattern. To indicate the quantitative protein expression profile of a cell, an organism, or a tissue under exactly defined conditions, analyze and compare protein expression profiles need proteomics tools, also proteomics analysis can link the observed protein pattern changes to the causal effects (69). 

Because of being disease processes and treatments at the protein level, proteomics could help develop pharmaceutical industry. Drugs affect protein expression and change the protein pattern. These will give information about the mechanism of action, therapeutic or toxicological effects. Various drugs might be grouped according to their signaling cascades or metabolomics pathways. One of The main approach in proteomics is the biological phenotype identification or induction and the proteins subsequent deconvolution that modulate leading to the change in phenotype (68, 70). Optimizing lead compounds for clinical development, characterizing drug effects and understanding protein toxicology, discovery and validation of drug are achieved by using proteomics study and analysis (71). 

Proteins are the most important class of molecules targeted that are remained by pharmacological agents. The proteins complex role in normal and diseased phenotypes is explained by using new technologies in proteomics that are important part in the comprehensive, characterization and prioritization of proteins as drug targets. In addition, the design of phenotypic screens can predict human disease and discover novel proteins which are accessible to therapeutic. 

*Target identification*

A critical success factor in the pharmaceutical industry is the choice of the right drug for the right target. Proteomics study of the correlation between proteome expression and physiological changes related to a healthy or diseased condition and drug mechanism can describe disease mechanisms and side effects. Also these studies can help to find new diagnostic markers and identify potential therapeutic targets (71). Understanding the modulation pathway of gene to phenotype at the molecular level and phenotypic consequences develop therapeutic strategy (72).

A gene can be transcribed into several mRNAs and subsequently mRNA translates into proteins. Proteins may be post-translationally modified and leaded to multiple forms that may have distinct functional roles. The stages in the pathway from DNA to RNA to protein can be basis for target identification. Inhibition of biological functions and result in a certain phenotype is the desired effect of the drug. However, a novel drug effect is the molecular modulation or inactivation of a protein, it is necessary to validate the target’s function at the protein level not at the mRNA level. There are complex targets when proteins have multiple structural and functional domains. Targeting the appropriate molecule to the specific functional epitope will improve success rate of drug discovery (70).

By association some technologies such as chemical genetics, the use of blocking antibodies or aptamers, analog-sensitive enzyme alleles, and chromophore-assisted laser inactivation (CALI) with functional proteomics, a direct link to the protein’s role in the physiology of the cell or organism are provided. Protein function can be determined not only by examination of the individual components of a cell, but also by localization and interaction protein partners in the context of individual cell type. The parallel evaluation of cellular and molecular protein function allows to make efficient decision for target identification and validation and lead drug discovery (73). 

Today, more than 95% of drugs in the market are proteins target (74), whereas many of the small number of drugs are DNA target (toxic due to lack of specificity) (75) and a few drugs are RNA target e.g antisense drug targeting cytomegalovirus infections of the eye (76).

*Drug discovery*

Drug discovery is a highly competitive process. Identification of a disease-related protein target is not sufficient to drug discovery. It requires the understanding of the biochemistry and the regulation of an appropriate protein pathway or cascade the study of human genes and proteins identifies new drugs for the treatments of disease. Drug discovery includes target identification and validation, drug screening and optimization. In absorption, distribution, metabolism and excretion (ADME) and toxicology laboratories, characterization of ADME, and toxicity of a drug candidate molecule is evaluated. Chemical, clinical and functional proteomics, posttranslational modification and protein complex analysis contribute to target identification and validation in drug discovery (77). Functional proteomics approaches explore the components and interactions of pathways and networks which lead to a disease condition. This detailed knowledge of pathways and networks are deranged can help in identification of new drug-targets. Chemical proteomics approaches aiming at the comprehension of drug interactome contribute to optimization of lead compounds to improve drug selectivity and specificity, thus reduce side effects (78).

**Figure 1:**
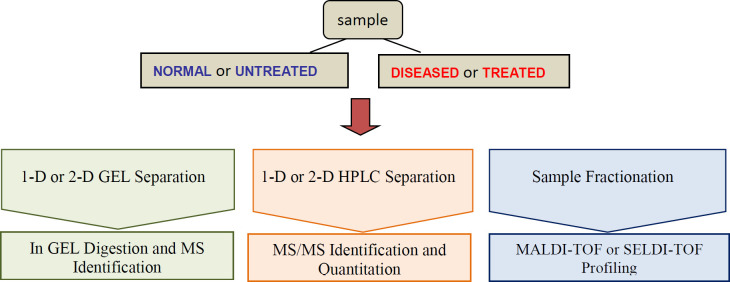
A partial view of proteomics approaches in clinic

The application of proteomics to human diseases study and their translation to the clinic has opened new horizons for medicine. The comparison of protein expression between healthy individuals and patients is the most promising tools for the identification of biomarkers. Clinical proteomic studies aiming at biomarker discovery require a platform to identify sensitivity, accuracy, reproducibility and high sample throughput. The providing of extensive proteome coverage of complex samples is required to allow the detection of proteins whose concentration levels vary by several orders of magnitude. High-throughput MS-based profiling strategies, MALDI-TOF and SELDI for clinical samples such as tissue and bodily fluids present the rapid determination of molecular masses and the ability of screening with high-throughput small amounts of clinical samples for biomarker discovery (Figure 1) (79).

**Figure 2:**
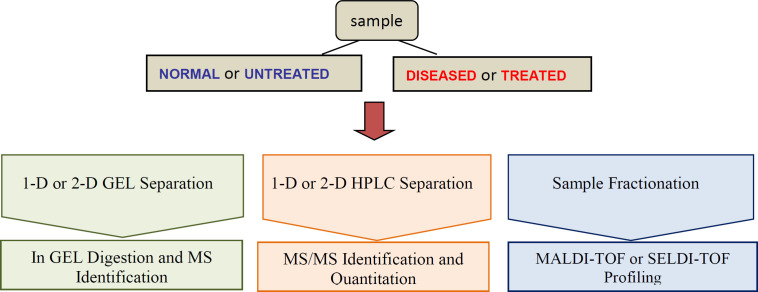
A partial view of Protein biomarker discovery

Proteomics technologies have been employed in the field of protein biomarker discovery. Progress in proteomics tools such as mass spectrometry (MS), selected reaction monitoring (SRM); multiple reaction monitoring (MRM) enable sensitive and accurate identification of thousands of proteins per experiment (80). These powerful tools are widely used to assess qualitative and quantitative differences in protein profiles of different samples, in particular diseased *vs. *normal and measure disease and drug related proteins. Proteomics in clinic discover, identify and quantify novel therapeutic biomarkers through assessing protein expression profiles and post translational modifications (PTMs) in healthy and diseased samples, or drug-treated samples (81) so early detection, diagnosis and therapeutic intervention of disease are facility. These biomarkers, as molecular targets, could provide valuable information for drug discovery (Figure 2). If the right therapeutic strategy is selected at an early stage of drug discovery process, time will significantly save, costs will reduce and the success rate of drug development will improve(69). 

There are two strategies for drug discovery: target-based drug discovery and phenotype-based drug discovery (82). In target-based drug discovery first, a target molecule is selected before screening. Target selection is based on its known role in disease or on the correlation of gene copy number, mutational status, or levels of RNA or protein with disease status. Then it is validated and in the development stage, it is followed by compound screening. The target proteins are usually screened against a large library of test compounds by using their enzymatic activities and establishing target selectivity such as detection of bounded, inhibited and activated proteins by the target molecule. In target-based strategy, using panels of enzyme assays for kinases, proteases, G protein-coupled receptors (GPCRs), ion channels, P450 enzymes, etc. can be achieved, but unexpected drug-target interactions cannot be identified (83). Target-based strategy as robust and cost-effective drug discovery method compared to phenotype-based strategy, in recent decades, is successful to advances in genomics and molecular biology.

In phenotype-based drug discovery focus on several cellular phenotypes such as cell proliferation (84) and differentiation (85), gene expression (86), protein expression (87) and phosphorylation (88), receptor translocation (85) and other mechanisms may be due to modulation of any protein within the probed signaling pathways without knowing of protein target and its responsibility for the observed effect (82). 

Term target deconvolution is used to describe proteomic experiments that identify the full spectrum of targets molecule associated with a bioactive molecule and the cellular phenotype. Target deconvolution needs to establish the efficacy target. Preclinical stages require a multitude of biochemical and genetic assays to characterize the effects of drug candidates on cellular systems and model organisms. By using proteomic techniques, protein targets identification is applied to elucidate the action mode of candidate drug molecules between scientific expectations and what the technology was able to deliver at the time. What a drug acts in the body such as drug efficacy, drug toxicity, and the therapeutic index of a drug must be checked in a relevant model system for that matter. Proteomics can be applied for Pharmacodynamics biomarkers identification in many forms.

A molecular pharmacodynamics biomarker should be able to monitor the intervention in a disease-relevant process or specific toxic effects in response to the treatment. the panel of in vitro and in vivo assays are established for a particular drug discovery project (83).

**Figure 3:**
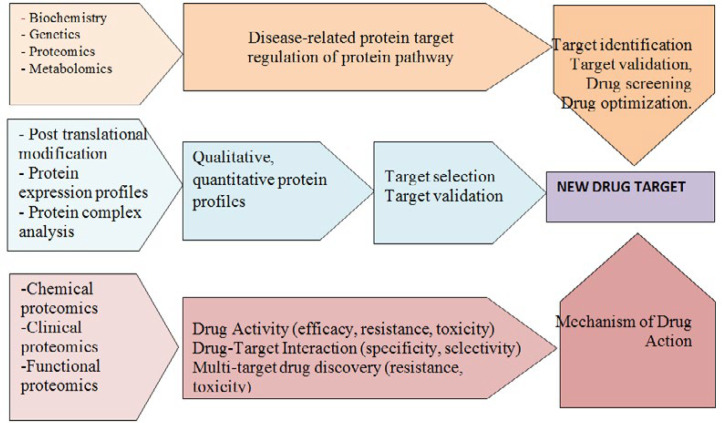
Applications of proteomics at target-based and phenotype-based drug discovery process

A variety of techniques and methods including in silico target prediction, genetic and transcriptional profiling, affinity-based techniques, yeast or mammalian three-hybrid systems, phage display, and chemo proteomics can be employed for these purpose (Figure 3) (89-91).

*Biomarker Discovery*

Proteomics characterize protein structure, function, protein-protein interactions, and associated protein modifications. Proteins with their characteristics collaborate to form complex signaling networks in the active cellular proteome. Proteomics output and patterns endpoints can be evaluated as biomarkers. Biomarkers are also evaluated as indicators of normal biological and pathogenic processes or pharmacological responses to a therapeutic intervention. It can be used to determine disease onset and progression, predict efficacy of treatment at a particular disease stage. These characteristics which are informative manors for clinical outcome can be categorized as prognostic or predictive biomarkers molecule. Prognostic biomarkers classify patients into subgroups such as progression or death; and predictive biomarkers classify patients into subgroups such as therapeutic sensitivity or resistance. Prognostic biomarkers cannot inform the choice of therapy (92). Understanding the active proteome is more important for development of effective predictive and prognostic biomarkers. identification of key potential events in the cellular proteome can be exploited for the development of targeted treatments (93, 94). Due to the availability of a large range of analytical instrumentation that can identify and quantify proteins in complex biological samples, protein expression profiling can be used to discover protein biomarkers and determine which combinations of them are most associated for early detection and prognosis of diseases. It can introduce markers for treatment response, prediction and monitoring. Biomarker discovery may provide potential markers for personalized patient therapy and monitoring of disease and can serve as possible drug targets (95-97). Some studies have established multiple predictive markers, but a few have been used in other populations as well for example, HER2 amplification in breast cancers (98) and ERCC1 in non-small cell lung cancer (99, 100) are applied as predictive biomarkers.

*Drug Toxicity*

Drug toxicity is a large proportion of failures in drug discovery and development. Drug toxicity accrues when a drug exhibits undesirable biological effects because it losses or gains of induced function so the useful treatment is limited. the p38 MAP kinase in inflammatory diseases is one of these toxic drug (101). Sometime, an unintended interaction of a drug with one or more proteins leads to adverse effects on the function of organs. The inhibition of drug-metabolizing enzymes of the P450 family (kinase inhibitors with limited selectivity) is one of these toxic drugs. Proteomic studies on drug selectivity can highlight potential toxicity issues early on and provide a valuable source for appropriate molecular toxicity biomarkers (102). Indeed, liver toxicity is frequently observed, imposing of the treatment effects is obtain by using global proteome profile of human hepatocytes or rodent livers exposed to a drugs. Proteins that are identified to change in abundance in response to drugs ,are useful surrogate pharmacodynamics biomarkers (103). 

imposing therapeutic effects obtained using the global proteomic profile of human liver or rodent liver exposed to a drug.

*Drug targets identification*

Proteomic studies play an important role in assessing protein structure, function, and cellular interactions. Chemical proteomics strategies can detect specific classes of proteins and represent an effective tool for isolation of subsequent protein from the proteome. As it is shown in the table 1, a variety of compound targets are discovered by using chemical proteomics, in particular, the targets of kinase inhibitors and natural products.

wide range of diseases, from cancers to autoimmune disorders develop from aberrant phosphorylation of protein kinases (104). A prevalent target of drug development is protein kinases. In recent years, dozens of kinase inhibitors have entered clinical trials, and many oral drugs have been used in clinics. The conserved ATP binding pocket is the most of these inhibitors target and has become a severe bottleneck compromising the clinical application of these drugs. Chemical proteomics identifies possible targets of kinase inhibitors by affinity purification from cellular extracts and resulting of whole spectrum identification of potential drug targets. It also provide a strong foundation for developing novel potent drugs without side effects (105). Pyrido [2,3d] pyrimidine is used as kinase inhibitor and its target profile is explored. Its targets, including Proto-oncogene tyrosine-protein kinase Src, platelet derived growth factor receptor (PDGFR) and fibroblast growth factor receptor (FGFR), Riplike interacting caspase like apoptosis regulatory protein kinase (RICK) and p38α are known (106). The serine/threonine type protein kinase C (PKC) family is a critical regulator in the activation of cellular functions and proliferation (107).

Bisindolylmaleimides, especially GF109203X , PKC inhibitor, have several targets including protein kinases CDK and Ste20-related, and non-protein kinases such as adenosine kinase and quinone reductase type-2. Also voltage-dependent sodium channels and the 5hydroxytryptamine 3 receptor act as potent targets of GF109203X (108, 109). Vascular endothelial growth factor receptor (VEGFR), PDGFR, and FGFR that are receptor tyrosine kinases (RTKs) play a critical role in endothelial cell growth and angiogenesis (113, 114). SU6668 , antiangiogenic small molecule drug, have wide range of targets such as VEGFR2 and βPDGFR(115), Ser/Thr kinase targets, including TBK1, Aurora kinases including RSK3 and AMPK (111). 

 Imatinib (116), nilotinib (117, 118) and dasatinib (119) as the well-known BCRABL tyrosine kinase inhibitor,and bosutinib (72) as SRC/ABL inhibitor are all used in clinic to treat chronic myeloid leukemia. By using affinity matrices in combination with liquid chromatography electrospray ionization tandem MS (LC-ESI-MS/MS), target profiles of imatinib, nilotinib, and dasatinib in K562 and CML primary cells are identifeid. The known major targets of imatinib are ABL, KIT, and PDGFR. 

A proteomics study by using ELISA and western blotting showed that macrophage colony stimulating factor receptor CFMS, a crucial regulator in the growth and differentiation of the monocyte macrophage lineage, is a novel target of imatinib (119, 120). Western blot analysis showed that imatinib didn’t influence CFMS protein expression but inhibits CFMS phosphorylation and inactives CFMS. In addition these results show that cABLn and BCRABL acted as interactors of nilotinib, ARG (ABL2), NQO2, and the receptor tyrosine kinase DDR1 (discoidin domain receptor 1) as significant targets of nilotinib are validated (116).

by using chemical proteomics partial targets of nilotinib, such as BCRABL, cABL, cKIT, and PDGFR are detected (117, 118). An in vitro study by using drug protein interaction profiles with chemical proteomic assays shows that dasatinib targeted a wide range of kinases and found cABL, BCRABL, and DDR1 were interacted with dasatinib. A study by LCMS/MS using total K562 cell lysates detects some potent proteins bind with dasatinib. These proteins include the TEC family kinases, BTK and TEC (119). 

*Drug development*

Drug development needs incorporating proteomics and biomarkers into clinical trials. Application of biomarkers and new identified drug targets required verification and biological validation in clinic. A key step in maximizing the clinical and commercial success of a biomarker is verification and validation of tests. Selection criteria to defined subsets of patients susceptible to specific targeted therapies are yielded by validated biomarkers. 

Proteomics studies can identify optimally targeted agent and biologically effective doses for each patient’s disease. It may be applied for monitoring of response and relapse. Also engineering of new drugs and understanding circumvent resistance mechanisms can be done by proteomics tools and studies. Creation a comprehensive database of information pairing genomic change with proteomic expression for cancer needs comprehensive systems biology proteomic approaches with agent’s target that derived from proteomics. It will be useful for patients and therapeutic. A clinical trial that based on biomarker strategy, will test the tumors of 10,000 patients with relapsed refractory cancer by genomic and proteomic technologies for patients are at high risk for recurrence with no therapeutic standard of care. Its results will identify the best treatment considerations (77).

**Figure 4:**
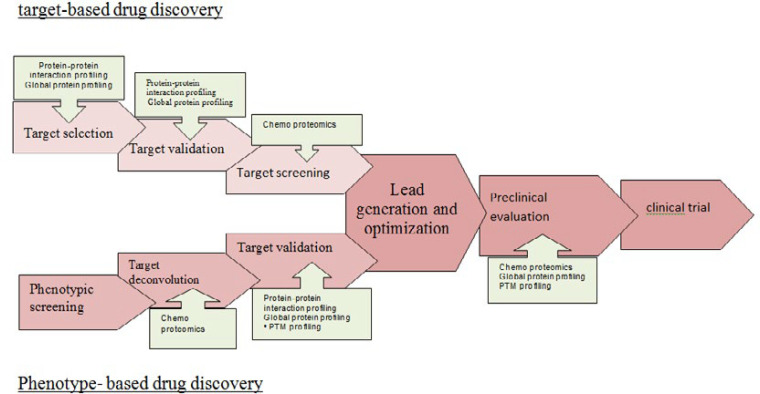
Application of proteomics as a tool of new biomarker and drug discovery

Proteomic technology is applied to targeted therapies and will bring to reality the clinical adoption of stratification of molecular proteomics. Proteomics profiling will be critical for development of biomarkers and potential drug targets. Proteomics studies will help dissect protein signaling pathways for definition of preferable targets of molecular therapy. A prospective validation study is an important method to guide future direction of proteomics. The discovery of novel, validated biomarker signatures will expand understanding diseases and will develop new potential drugs for more effective targeted therapy in recurrent cancers. In future, these events will guide directions of proteomics as a tool of new biomarker and drug discovery in diseases (Figure 4). 

**Table 1:** drug targets which are identified in cancer and Chronic myeloid leukemia by proteomics

disease	drug	Target	Proteomic tools
Cancer	Pyrido[2,3-d] pyrimidine (106)	Src, PDGFR, FGFR, RICK, p38α	Affinity chromatography Nano HPLC MS/MS LC-M S/MS
	GF109203X(108, 109)	PKC, Ste2-re lated kinase, adenosine kinase, quinine reductase type 2, voltage-de pendent sodium channels, 5-HT 3 receptor	SDS-PAGE separation immunoprecipitation MS
	Bisindolyl-Maleimide-III(110)	PKC-α , GSK3-β , CaMKII, adenosine kinase, CDK2, quinine reductase type2, PKAC-R , prohibitin, VDAC, heme binding proteins	Affinity chromatography, MS
	SU6668(111)	βPD GFR, VEGFR2, FGFR, Lyn, RSK3, AMPKα1 ,ULK3	16-BA C/SDS-PAGE Immunofluorescence, MS
Chronic myeloid leukemia	Imatinib	BCR-AB L, ABL, c-KI T, PDGFR, NQO2, c-fms	LC-ESI-M S/MS, HPL-M S, ELISA, western blotting
	Nilotinib	c-AB L, BCR-AB L, c-KIT, PDGFR, ARG, NQO2, DDR1	LC-ESI-M S/MS, HPLC-M S, immunoblotting
	Dasatinib	c-AB L, BCR-AB L, BCR-AB L, DDR1,BTK, TEC	LC-ESI-M S/MS immunoblotting, SDS/PAGE, LC-M S/MS
	Bosutinib(112)	ABL , SRC family kinases, STE, TEC family kinases, CAMK2G	Affinity chromatography, MS, kinobeads/iTRAQ

PDGFR, platelet-de rived growth factor receptor ; FGFR, fibroblast growth factor receptor; RICK, Rip-lik e interacting caspase-lik e apoptosis-regulatory protein kinase; CK1, Casein kinase 1; GAK, cyclin G-as sociated kinase; PKNβ, protein kinase N beta; JAK1, Janus kinase 1; NQO2,quinone oxidoreductase 2; DDR1, discoidin domain receptor-1; BTK, Bruton's tyrosine kinase; CAMK2G, calcium/calmodulin-de pendent protein kinase type II gamma chain; CDKs, cyclin-dependent kinases; PDXK, pyridoxal kinase; PKC, protein kinase C; VDAC, voltage-dependent anionchannel; AMPKα1 , the AMP-activated protein kinase -1 ; ULK3, Unc-51-lik e kinase 3; bFGF, basic fibroblast growth factor; CRBN, cereblon.

## Conclusion

Wide spread relationship between proteomics and advances in biomedical sciences indicates that proteomics as a fundamental technology is applied in the fields. Drug targets identification, drug discovery, drugs development, and drug toxicity are the main fields in the pharmaceutical industry that are affected by proteomics researches. Since biomarker discovery has a significant role in drug development and proteomics is a useful method for biomarker discovery, drug development progress is tied to advances application of proteomics. It is a logical conclusion that economy of pharmaceutical industry is affected by proteomics effectively
